# The complete nucleotide sequence of the mitochondrial genome of *Dorcadia ioffi* (Siphonaptera: Vermipsyllidae)

**DOI:** 10.1080/23802359.2017.1347901

**Published:** 2017-07-07

**Authors:** Hai-Tao Xiang, Feng-Qin Wen, Guo-Li Wang

**Affiliations:** a College of Veterinary Medicine, Gansu Agricultural University, Lanzhou, China;; b College of Plant Protection, Gansu Agricultural University, Lanzhou, China

**Keywords:** Mitogenome, *Dorcadia ioffi*, Siphonaptera, Vermipsyllidae

## Abstract

In this study, the complete mitochondrial genome of *Dorcadia ioffi* was determined. The mitogenome is 16,785 bp in length and contains 13 protein-coding genes, 22 tRNA genes, 2 rRNA genes, and 1 control region. The nucleotide composition of the *D. ioffi* mitogenome was A: 40.10%, T: 40.61%, G: 7.74%, C: 11.55%. The A + T content is 80.71%, showing strong AT skew. Phylogenetic analysis indicated that Siphonapteran may have sister relationship with Dipteran.

*Dorcadia ioffi*, belonging to the family Vermipsyllidae in the order Siphonaptera, is an blooding-sucking ectoparasite of domestic animals (sheep especially). This flea has been discovered in China for a long time.

In our study, one female adult flea was used to determine the complete mitochondrial DNA, which was collected from Tibetan sheep in the Northwest of China. Other insects were packed in 100% ethanol and stored in Veterinary Medicine Museum of Gansu Agricultural University. Next-generation sequencing (NGS) technology was used to obtain the complete mitogenome of the sheep flea, *D. ioffi*.

The complete mitochondrial genome of *D. ioffi* is a closed circular molecule 16,785 bp in length (Genebank accession number MF124314), shorter than the mitogenome of *Jellisonia amadoi* (Cameron 2015). The nucleotide composition of the *D. ioffi* mitogenome was A: 40.10%, T: 40.61%, G: 7.74%, C: 11.55%. The A + T content is 80.71%, showing AT skew, which is consistent with the characteristics of the nucleotide composition of most insects (Arnemann et al. 2016; Zhang et al. 2017). The PhyML 3.0 web server (Guindon et al. 2010) was used to infer the relationship between *D. ioffi* and other blood-sucking insects (Figure 1). The results showed that *D. ioffi* and *Jellisonia amadoi* gather together as one branch, because they belong to the order Siphonaptera. From an overall point of view, phylogenetic analysis indicated that Siphonapteran may have sister relationship with Dipteran.

**Figure 1.**
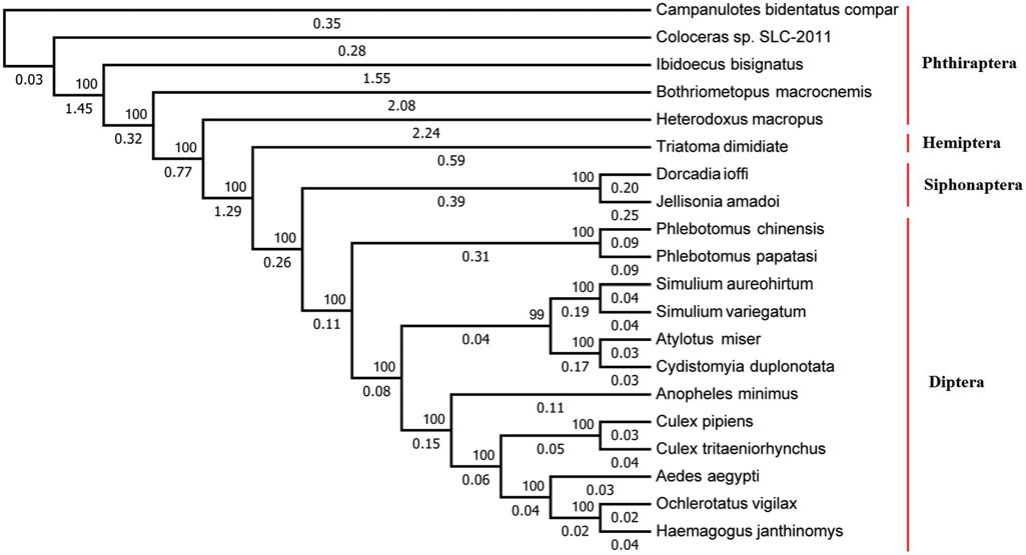
Phylogenetic analysis of 20 blood-sucking insects using amino acid sequences of 13 protein-coding genes of mitogenome. Data sources: *Campanulotes bidentatus compare* (AY968672); *Coloceras* sp. SLC-2011 (JN122000); *Ibidoecus bisignatus* (JN122005); *Bothriometopus macrocnemis* (EU183542); *Heterodoxus macropus* (AF270939); *Triatoma dimidiate* (AF301594); *Dorcadia ioffi* (MF124314); *Jellisonia amadoi* (KF322091); *Phlebotomus chinensis* (KR349297); *Phlebotomus papatasi* (KR349298); *Simulium aureohirtum* (KP793690); *Simulium variegatum* (KU252587); *Atylotus miser* (KT225291); *Cydistomyia duplonotata* (DQ866052); *Anopheles minimus* (KT895423); *Culex pipiens* (KT851543); *Culex tritaeniorhynchus* (KT851544); *Aedes aegypti* (EU352212); *Ochlerotatus vigilax* (KP721463); *Haemagogus janthinomys* (KT372555).
